# The Effect of Doxapram on Proprioceptive Neurons: Invertebrate Model

**DOI:** 10.3390/neurosci3040041

**Published:** 2022-10-23

**Authors:** Bethany J. Ison, Maya O. Abul-Khoudoud, Sufia Ahmed, Abraham W. Alhamdani, Clair Ashley, Patrick C. Bidros, Constance O. Bledsoe, Kayli E. Bolton, Jerone G. Capili, Jamie N. Henning, Madison Moon, Panhavuth Phe, Samuel B. Stonecipher, Hannah N. Tanner, Logan T. Turner, Isabelle N. Taylor, Mikaela L. Wagers, Aaron K. West, Robin L. Cooper

**Affiliations:** Department of Biology, University of Kentucky, Lexington, KY 40506, USA

**Keywords:** crab, K2p channels, proprioception, sensory

## Abstract

The resting membrane potential enables neurons to rapidly initiate and conduct electrical signals. K2p channels are key in maintaining this membrane potential and electrical excitability. They direct the resting membrane potential toward the K^+^ equilibrium potential. Doxapram is a known blocker for a subset of K2p channels that are pH sensitive. We assessed the effects of 0.1 and 5 mM doxapram on the neural activity within the propodite-dactylopodite (PD) proprioceptive sensory organ in the walking legs of blue crabs (*Callinectes sapidus*). Results indicate that 0.1 mM doxapram enhances excitation, while the higher concentration 5 mM may over-excite the neurons and promote a sustained absolute refractory period until the compound is removed. The effect of 5 mM doxapram mimics the effect of 40 mM K^+^ exposure. Verapamil, another known K2p channel blocker as well as an L-type Ca^2+^ channel blocker, reduces neural activity at both 0.1 and 5 mM. Verapamil may block stretch activated channels in sensory endings, in addition to reducing the amplitude of the compound action potential with whole nerve preparations. These findings are notable as they demonstrate that doxapram has acute effects on neurons of crustaceans, suggesting a targeted K2p channel. The actions of verapamil are complex due to the potential of affecting multiple ion channels in this preparation. Crustacean neurons can aid in understanding the mechanisms of action of various pharmacological agents as more information is gained.

## 1. Introduction

The resting membrane potentials of most cells are maintained by K^+^ leak channels, also known as K2p channels (two-pore domain K^+^ channels). They were first found in yeast and have since been identified in the genomes of various organisms from plants to humans [1,2,3,4]. These channels are active in an open state, allowing K^+^ ions to flux, driving the resting membrane potential toward the K^+^ equilibrium potential.

K2p channels are classified based on their structures and sensitivities. There are 15 known types of K2p channels in mammals. They are grouped into six subtypes with varying pharmacological profiles and distribution in various tissues. The channels show subtype selectivity to volatile anesthetics, pH, membrane tension, endocannabinoids, hypoxia, heat, and G protein-coupled receptor agonists [5,6,7]. Some compounds, such as chloroform, halothane, and isoflurane [8], stimulate a subtype of K2p channels resulting in the hyperpolarization of cells. The subtype K2P13 is activated by arachidonic acid [9]. Some channels (i.e., K2P18) can be activated by volatile anesthetics (e.g., isoflurane, sevoflurane, halothane, desflurane) but are also reduced in function by local anesthetics (e.g., including bupivacaine, tetracaine, ropivacaine, mepivacaine, lidocaine [7]), leading to the depolarization of cells.

Doxapram, also known by the trade names Stimulex and Respiram, is an inhibitor of two K2p channels: TASK1 and TASK3, which are in the TASK (TWIK-Related Acid-Sensitive K+) channel subtype [6,10]. Since these channels are present in carotid bodies, doxapram can be used clinically to stimulate associated neurons to enhance respiratory drive when inducing therapeutic hypothermia [11,12,13] or for treatments of respiratory disorders and apnea in infants [14,15].

The actions of pharmacological agents like doxapram have not been fully examined for potential effects on other cellular functions. Agonists and antagonists of K2p channels may impact many cell types yet to be investigated. Clinically, this is important to understand, as potential undesirable effects may arise by action on other cell types [16,17,18]. Experimental animal models can aid in screening novel pharmacological agents targeting subtypes of K2p channels. Verapamil, commonly known as a Ca^2+^ channel blocker, was recently shown to inhibit a K2p subtypes known as K_2P_18.1. Learning how K2p channels function and what drives expression of their subtypes may help to understand why the expression profiles vary in disease states, such as cancer [18] and forms of epilepsy [19]. In addition, addressing pharmacological agents used in mammals in other organisms aids in understanding their mechanisms of action.

Invertebrate models have assisted in the advancement of neurophysiology. They are inexpensive and easy to obtain, but also remain stable in simple saline for conducting experiments at room temperature. These models also have regions of neurons (i.e., soma and axons) large enough for intracellular recordings. Squid, crustaceans, and insects are commonly used to address fundamental concepts in physiology and in particular neurobiological topics [20,21,22,23,24,25].

The blue crab (*Callinectes sapidus*) has recently been used to investigate the effects of extra- and intra-cellular pH on neuronal excitability of sensory neurons [26]. Various aspects of the neural circuitry of the crab, *Cancer borealis*, were shown to respond differentially to extreme differences in extracellular pH. Responses mediated by pH may function via K2p channels expressed in neurons of crab models. Such responses have yet to be examined in genomic analysis or physiologically for acid-sensitive K2p channels, such as the doxapram-sensitive TASK channels. This study was exploratory to determine if sensory neurons respond to doxapram and how the compound alters the activity profile.

## 2. Materials and methods

### 2.1. Animals

The preparations used for this study were wild-caught blue crabs (*Callinectes sapidus*) from August and October 2022. They were purchased at a local supermarket in Lexington, KY, USA, which originated from a distribution center in Atlanta, GA, USA. Crabs were all female adults in the range of 10–15 cm in carapace width (from point to point). They were maintained in a seawater aquarium for several days prior to use to assess their health. Upon examination and muscle dissection, some specimens had cysts, while others had missing limbs. All crabs used were alive and active upon autotomizing a leg for experimentation.

### 2.2. Chemicals and Dissection

The physiological saline used to obtain recordings and maintain preparations consisted of (in mM): 470 NaCl, 7.9 KCl, 15.0 CaCl_2_·2H_2_O, 6.98 MgCl_2_·6H_2_O, 11.0 dextrose, HEPES acid and 5 HEPES base normalized to pH 7.5. Doxapram powder was added directly to the saline to obtain 0.1 mM or 5 mM concentrations and placed on a vortex (high setting) for 5 min. The saline solution remained opaque for the 5 mM solution. Verapamil hydrochloride was directly dissolved in the crab saline at 0.1 and 5 mM. All compounds were obtained from Sigma-Aldrich (St. Louis, MO, USA).

The dissection to expose the propodite-dactylopodite (PD) organ in the walking legs and to record from the transected PD nerve with a suction electrode is detailed in Pankau et al. [27], and a detailed movie is provided in Majeed et al. [28]. Crabs were induced to autotomize the first or second walking leg by using forceps to apply gentle pressure at the coxa of the limbs. Forceps were used to gently pull the PD nerve away from the primary leg nerve under a dissecting microscope. The proximal aspect of the leg was then secured with insect dissection pins in a Sylgard-lined dish filled with crab saline, and the isolated PD nerve was placed in a suction electrode to measure its activity.

Procedures similar to the PD nerve isolation and compound action potential (CAP) recording techniques described by Pankau et al. [27] were followed. In brief, the PD nerve was isolated by dissecting away the main leg nerve. At the distal end of the PD nerve, a stimulating electrode was placed to induce CAPs. The proximal portion of the nerve extending from the propodite region was placed in a recording electrode to measure the induced CAPs. A ground wire was placed along the side of the dish. Following electrode placement, both the distal and proximal ends of the nerve were packed with petroleum jelly to increase contact between the electrode and nerve surface, therefore maximizing the amplitude of the extracellular signals recorded. The nerve was stimulated until the voltage producing maximum CAP amplitude and duration was identified. With the maximum CAP amplitude and duration serving as a point of reference, any change in the amplitude or area under the trace of the CAPs could be clearly identified upon exposure to the various solutions.

### 2.3. Data Collection

Treatment groups consisted of two concentrations (0.1 mM and 5 mM) of doxapram or verapamil. For each concentration, six preparations were first exposed to a saline control, then to doxapram or verapamil, then incubated in doxapram or verapamil for 5 min and the activity was assessed. Preparations were incubated for another 15 min (20 min total) and assessed again for activity, after which two saline washes occurred. The preparations were retested to see if changes in neural activity due to the pharmacological compounds were reversible. To confirm the experimental findings, participants in a university course tested five more preparations with 5 mM doxapram. Experiments were also conducted in six different preparations where the saline bath was exchanged with 20 mM KCl followed with 40 mM KCl and a saline wash out.

**Figure 1:**
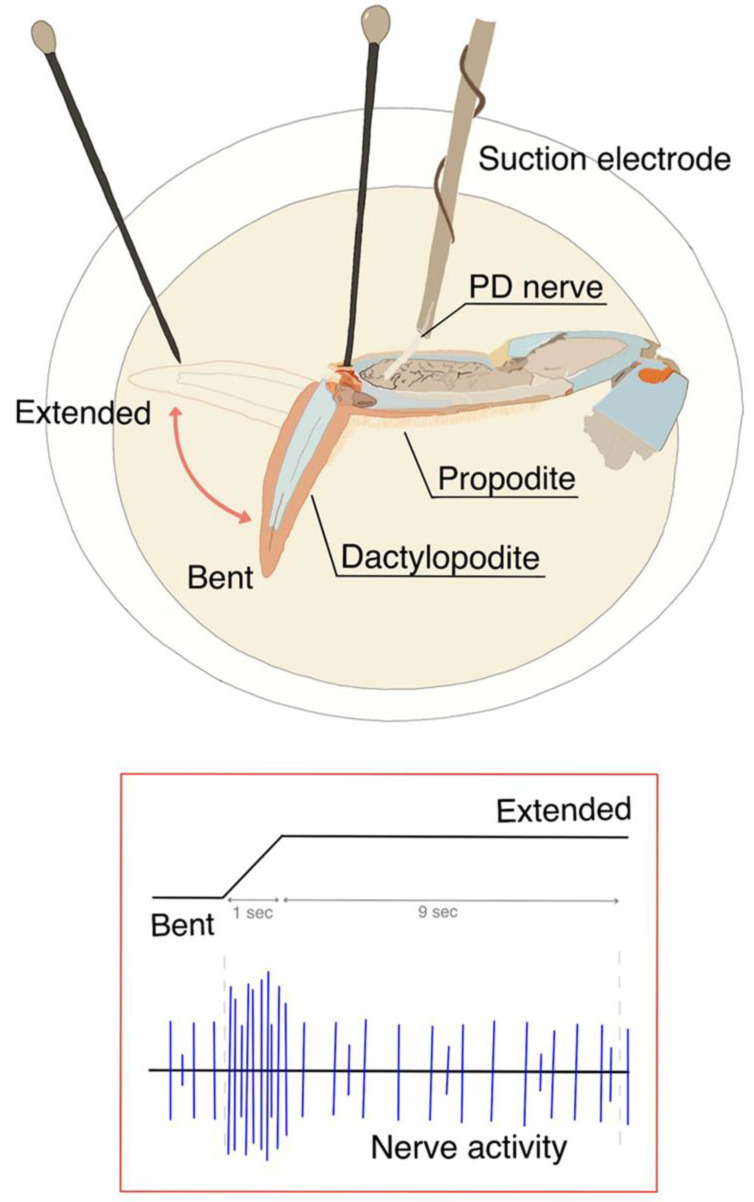
The first or second walking leg of the crab was used to expose the PD organ and associated nerve to various compounds. The joint was initially bent at 90 degrees, then extended out straight within 1 s, and then held for at least another 9 s. The entire 10 s was then used for analysis in the number of spikes that occurred while bathed in different solutions.

For each condition, the dactyl (most distal segment of the leg) was moved from a flexed position of 90 degrees to full extension in 1 s and then held in the extended position for at least 9 more seconds. The joint was then returned to the 90-degree bend (Figure 1) All 10 s of data recordings were used during analysis. Throughout each trial, a dissecting pin was used as a stop point to ensure consistency in the maximum range of extension obtained.

### 2.4. Analysis of the Neural Activity

Spikes were counted in the 10-s window during the movement and time held in the extended position via the software in Chart 7 and Chart 8 (ADI Instruments) (Figure 1). A sine wave fit of the trace was selected, and standard deviation was set to ensure proper signal detection. The amplitudes of spikes had a wide range from large to small; the choice of which spikes to count was determined by eye and was approximately the amplitude of two times the baseline noise.

The number of spikes for each trace was determined by one individual to maintain consistency in analysis. The software does produce errors in determining the signals from noise. Therefore, counts were confirmed by eye and adjusted as needed by modifying the standard deviation within the software. Software issues were detailed in a previous report [29]. To examine reproducibility in the analysis, the university students were provided samples of the same data sets to compare to the original analysis.

### 2.5. Statistical Methods

A paired T-test or a Wilcoxon Signed Rank Test and a significance level of 0.05 was used in all studies. This study was also conducted, in part, with participants in a university senior-level neurophysiology course as part of an ACURE (Authentic Course-based Undergraduate Research Experience) [26,27,28,29]. This helped confirm the experimental findings and the reproducibility of the data analysis.

## 3. Results

### 3.1. Effects of Doxapram on Neural Activity

**Figure 2:**
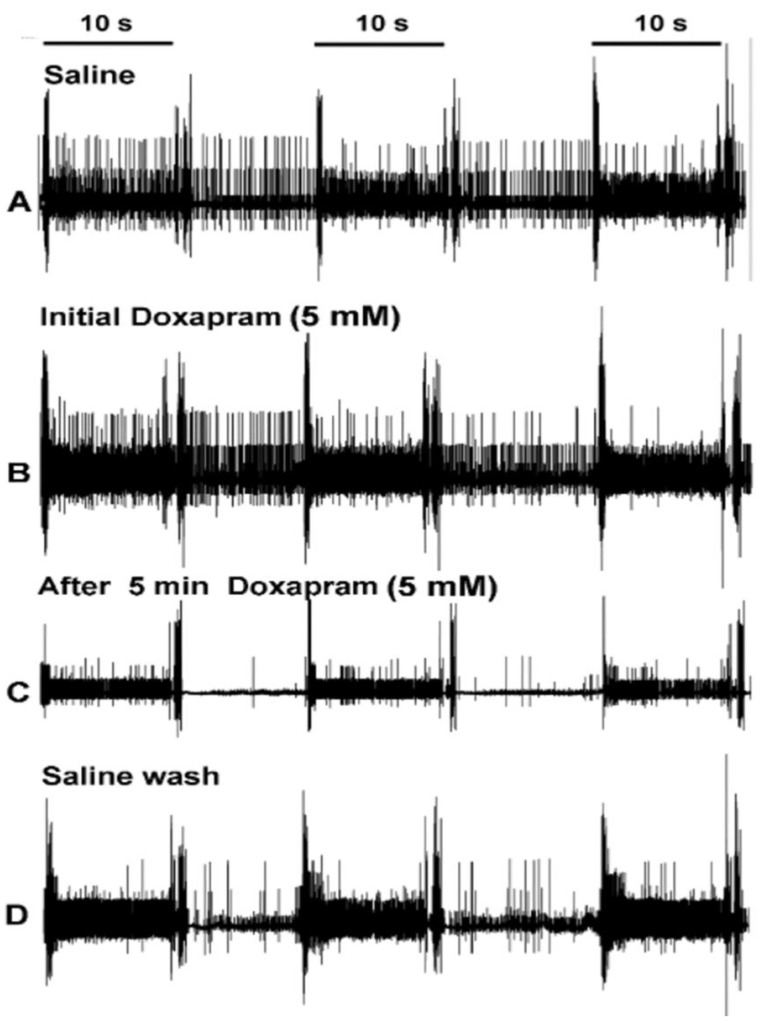
Representation of the effects of doxapram at 5 mM on neural activity for the proprioceptive neurons in the crab PD organ. (**A**) The activity of the nerve in saline with the three movements of the joint (1 s for the movement to an extended position and 9 s or more for being held in a static position of joint extension). (**B**) After the bath is exchanged to doxapram, the joint is then moved again three times. (**C**) Flushing the doxapram solution around the preparation and allowing it to incubate for 5 min. After 5 min, three more movements are made. (**D**) The bath is exchanged two times with fresh saline and the joint movements are repeated. Only the initial 10 s are used for analysis.

To examine the effect of doxapram at 5 mM on neural activity during movements of the PD joint, the number of spikes were counted (Figure 2). A representative preparation is illustrated in Figure 2 with the initial saline exposure (Figure 2A) followed by a bath exchange to 5 mM doxapram (Figure 2B). The bath was flushed and allowed to incubate for 5 min before repeating three more joint movements (Figure 2C). Afterward, the bath was exchanged with fresh saline which was flushed around the preparation. Three more trials were then repeated (Figure 2D).

**Figure 3:**
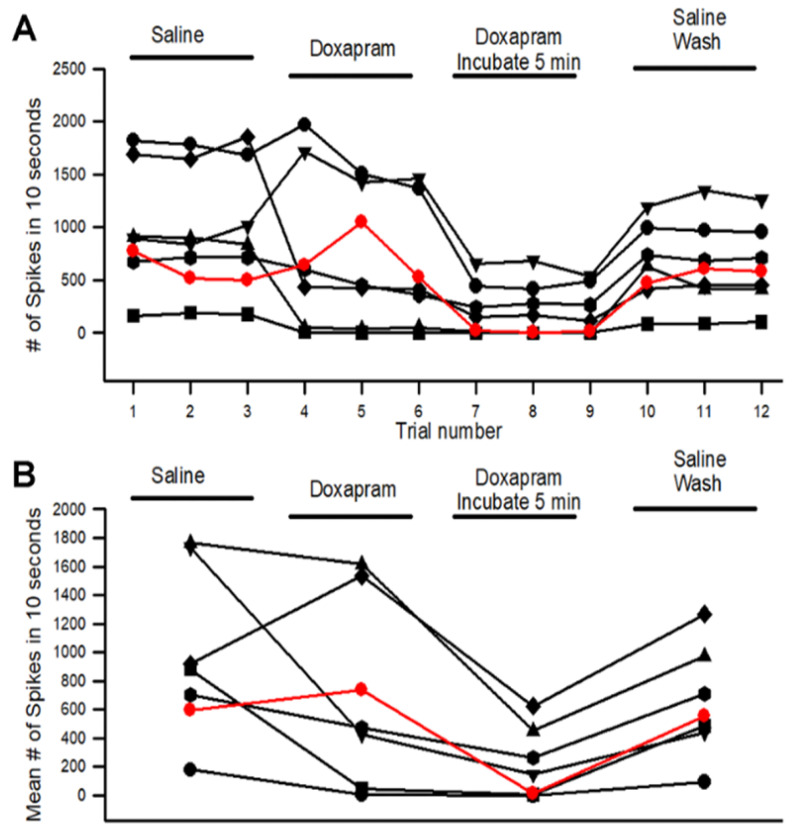
The acute effect of doxapram (5 mM) on neural activity of the PD organ. (**A**) The number of spikes measured in the 10-s window from the beginning movement of the joint starting from a bent position (90 degrees) to fully extended within 1 s and held in an extended position for the next 9 s. This paradigm is repeated three times for each condition. Each line represents a different preparation of an PD organ. Three trials were undertaken with saline, three trials were done immediately after switching the bath to doxapram (5 mM), and they were examined again after incubation for 5 min. The final exchange was to rinse the preparation twice with fresh saline and then move the joint three more times. Each movement was separated by at least 10 s while the joint was held in a bent position. (**B**) The number of spikes in each of the three trails was averaged and graphed in the same manner as in (**A**), which allows an easier view of the overall effects. The red colored trace represents a PD preparation from a chela of the large claw.

The activity for the three trials in each bathing condition from the 6 walking legs and 1 large cheliped is shown in Figure 3A. The data obtained from the large cheliped is shown in red to separate the responses from the walking legs. However, the overall results fall within the range obtained for the walking legs. The mean number of spikes obtained in each of the three trials for each condition is shown in Figure 3B. There is a significant decrease in activity with incubating the preparation for 5 min in doxapram (5 mM) (*p* < 0.5 Paired T-Test, N = 7).

To examine reproducibility in the effect of doxapram at 5 mM independently of the data presented above, 17 participants in a university senior-level neurophysiology course repeated the experiments in five groups. The results of the classroom experiments are shown in the Appendix A.

In addition, data sets were provided to the class to analyze the number of spikes. The conditions of the data were blind to the participants. One trained individual analyzed all data sets in this study for all experiments. The results in reproducibility in data analysis are presented in the Appendix A.

**Figure 4:**
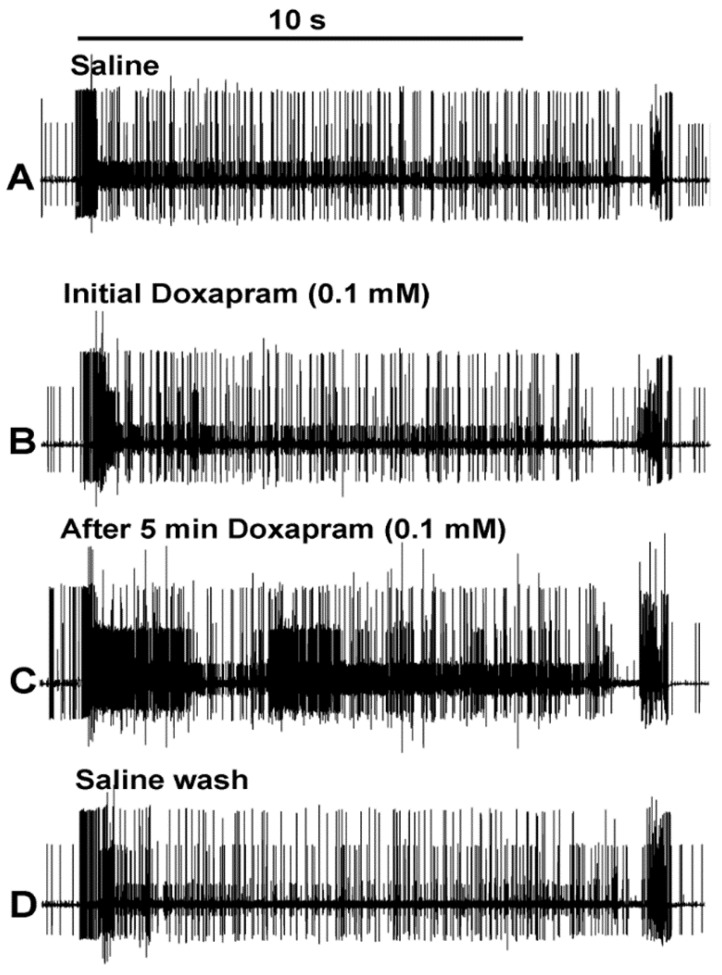
Representative responses to the effects of 0.1 doxapram exposure. The second trial of the three movements for each condition: (**A**) initial saline, (**B**) initial exposure to doxapram, (**C**) 5 min of incubation to doxapram, and (**D**) saline wash out. The number of spikes within the initial 10 s is used for quantification.

Since the number of spikes in three preparations first demonstrated an increase in activity before decreasing with the initial exposure, it was assumed that the initial exposure might take some time for the full effect. One reason the preparations were incubated for 5 min while flushing the bath was to allow the preparations to be well exposed. The blocking of K2p channels would depolarize the neurons and potentially inactivate the ability of the nerve to have repetitive firing. Thus, a lower concentration was used at 0.1 mM with flushing the bath well around the preparation as well as allowing 5 min of incubation time as performed for the 5 mM concentration. The neural activity from a representative preparation is shown in Figure 4. The second trial in each condition (A-Saline; B-Doxapram; C-After 5 min of incubation in doxapram; D- Saline wash) is shown. Illustrating the enlarged second trial of the three for each condition allows one to readily see the differences in the number of individual spikes for each condition.

**Figure 5:**
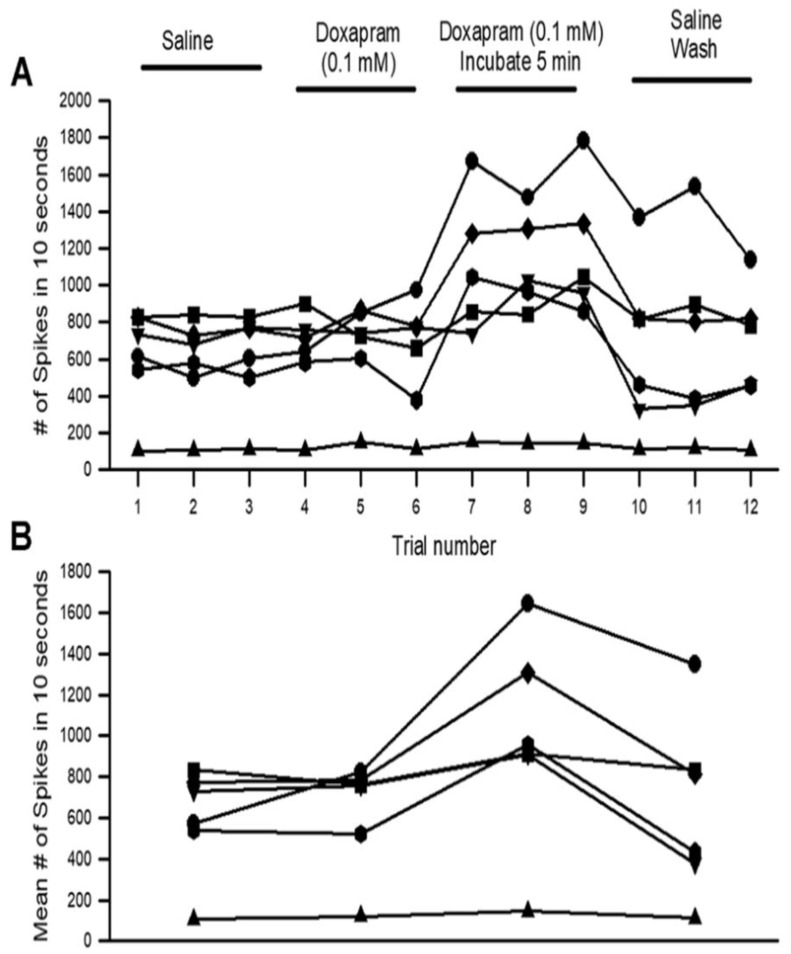
The acute effect of doxapram (0.1 mM) on neural activity of the PD organ. (**A**) The number of spikes measured in the 10 s window from the beginning movement of the joint starting from a bent position (90 degrees) to fully extended within 1 s and held in an extended position for the next 9 s. This paradigm is repeated three times for each condition. Each line represents a different preparation of a PD organ. Three trials were undertaken with saline, three trials were done immediately after switching the bath to doxapram (0.1 mM), and they were examined again after incubation for 5 min. The final exchange was to rinse the preparation twice with fresh saline and then move the joint three more times. Each movement was separated by at least 10 s while the joint was held in a bent position. (**B**) The number of spikes in each of the three trials was averaged and graphed in the same manner as in (**A**), which allows an easier view of the overall effects.

The number of spikes varied among the preparations (Figure 5A), but the overall trend showed an increase in the number of spikes after the 5-min incubation. This was readily observed in the mean of the three trials for each condition (Figure 5: *p* < 0.05; paired T-Test; N = 6).

### 3.2. Effects of Raised Extracellular K^+^ on Neural Activity

**Figure 6:**
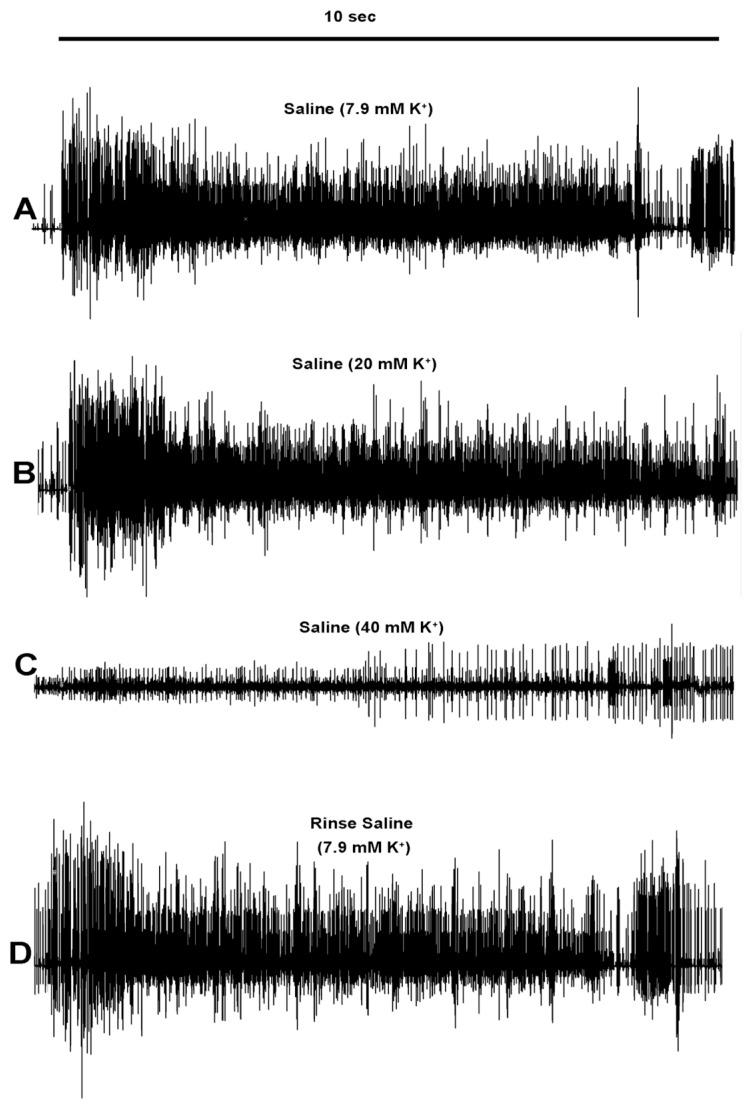
The effect of raised extracellular K^+^ on the neural activity for a representative PD organ. The activity of the PD nerve over 10 s when the joint from a 90-degree angle is fully extended within 1 sec and held for 9 s. The activity in saline (**A**) to saline containing 20 mM K^+^ (**B**). The 20 mM K^+^ did not show a significant change in overall activity. A change to a saline with 40 mM K^+^ (**C**) decreased the activity substantially. Some activity is regained with bathing the preparation in fresh saline (**D**).

**Figure 7:**
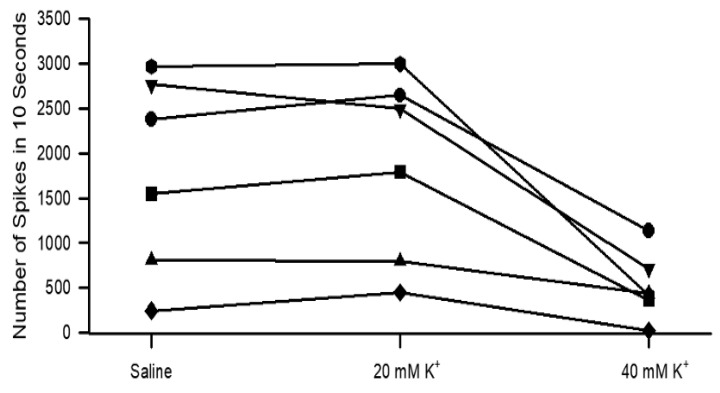
The number of spikes within 10 s when displacing the joint and holding it in a static position for six different preparations. There is no a significant effect for exposure to 20 mM K^+^ but there is a significant decrease in activity when exposed to 40 mM K^+^ (*p* < 0.05 paired T-Test).

To address the possibility that doxapram depolarized the neurons to an unexcitable state by depolarizing the membrane and inactivating the voltage-gated sodium channels, the PD organ was exposed to a higher-than-normal concentration of extracellular K^+^. The concentration of KCl of 40 mM rapidly depressed the neural activity of the PD nerve during the joint movements (Figure 6). Upon exchanging the bathing media back to saline, the neural activity was able to return (Figure 6D and Figure 7).

### 3.3. Effects of Verapamil on Neural Activity

Verapamil has recently been described to inhibit a specific subtype of K2p channels (i.e., K_2P_18.1) in mammalian neurons, and is a specific L-Type Ca^2+^ channel blocker in cardiac and neuronal tissues [30,31] For comparison to the effects of doxapram, the same concentrations were examined for verapamil (0.1 and 5 mM). The action of the in-situ PD organ and the excised nerve to examine the effect on the compound action potential (CAP) independent of the sensory endings with the stretch activated ion channels was examined.

**Figure 8:**
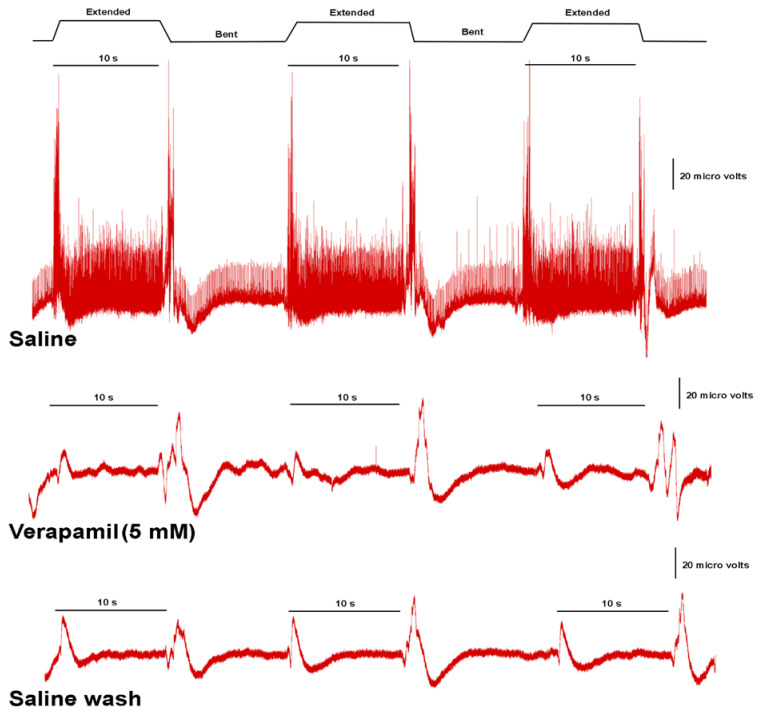
A representative effect of verapamil (5 mM) on neural activity of the PD nerve during the extension and bending of the PD joint. The movement of the joint is shown at the top and the 10 s used for analysis in the number of spikes recorded are shown for each paradigm. Three trials of movement are used initially during saline and when switching the bath to verapamil and following the flushing of the recording chamber with fresh saline.

**Figure 9:**
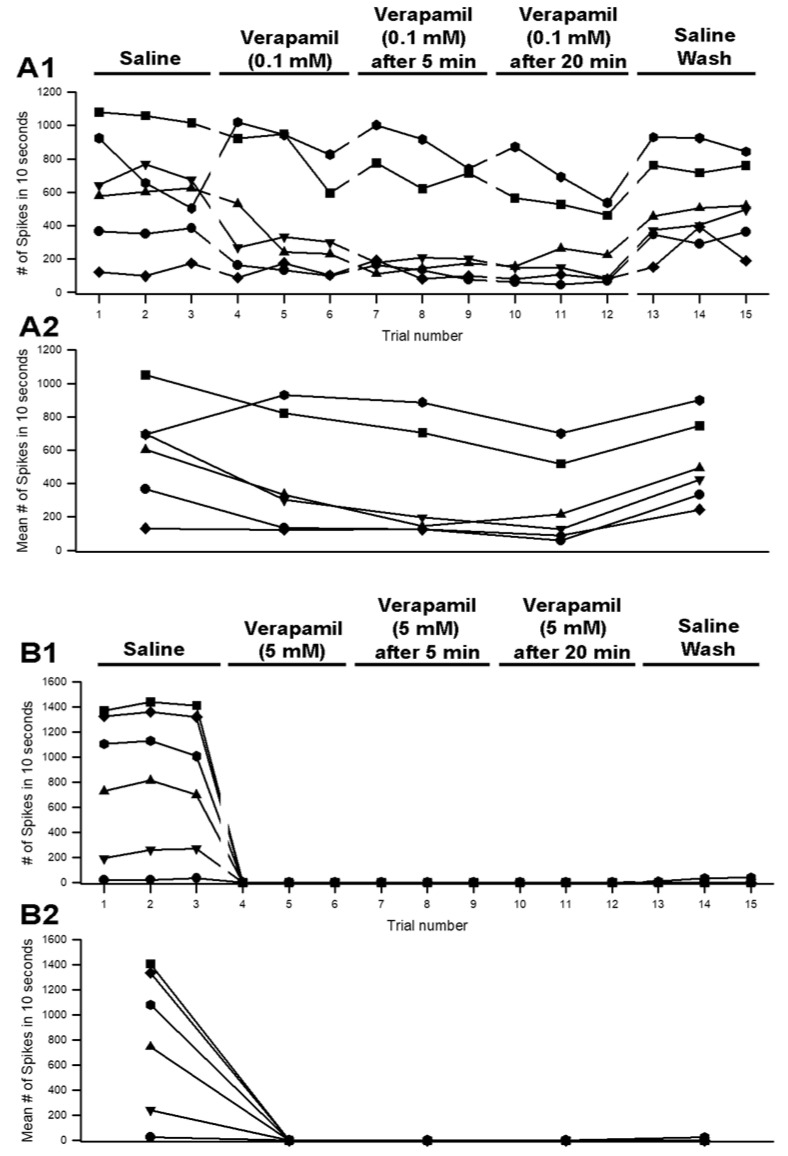
The acute effect of verapamil (0.1 mM and 5 mM) on neural activity of the PD organ. (**A1**) The number of spikes measured in the 10 s window from the beginning movement of the joint starting from a bent position (90 degrees) to fully extended within 1 s and held in an extended position for the next 9 s. This paradigm is repeated three times for each condition. Each line represents a different preparation of a PD organ. Three trials were undertaken with saline, three trials were done immediately after switching the bath to verapamil (0.1 mM), and they were examined again after incubation for 5 and 20 min. The last step was to rinse the preparation with fresh saline twice and then move the joint three more times. Each movement was separated by at least 10 s while the joint was held in a bent position. (**A2**) The number of spikes in each of the three trials was averaged and graphed in the same manner as in (**A1**), which allows an easier view of the overall effects. (**B1**) The same analysis is shown for the three trials in each condition for exposure to 5 mM verapamil. (**B2**) The average of each of the three trials for each condition is shown. Depression in neural activity was present at 0.1 and 5 mM after 20 min (*p* < 0.05; paired T-Test; N = 6 for each concentration). However, activity was depressed even after the initial exposure to 5 mm (*p* < 0.05; paired T-Test; N = 6).

A representative preparation for the effect of verapamil at 5 mM on neural activity while moving the PD joint is shown (Figure 8). We used the same paradigm for the exposure with doxapram by bending the joint and extending it three times in each bathing environment (saline, verapamil, saline wash). A total of 10 s were used for the analysis. This consisted of the number of spikes in the first 1 s it took to extend the joint and the subsequent 9 s while the joint was extended. Figure 9 illustrates the number of spikes among the six preparations exposed to 0.1 mM (Figure 9(A1)) and 5 mM (Figure 9(B1)). The activity in saline and initially after changing the bathing media to verapamil, as well as after five and 20 min of incubation, was used for quantification. After vigorous rinsing of the preparations with fresh saline, after exposure to 5 mM verapamil, the activity did not return. After the 0.1 mM exposure and rinsing, the neural activity did partially return. Both 0.1 mM and 5 mM resulted in a significant reduction in neural activity after 20 min of incubation (*p* < 0.05; paired T-Test; N = 6 for each concentration). A more rapid and prominent effect when compared to doxapram at the same concentrations was observed.

**Figure 10:**
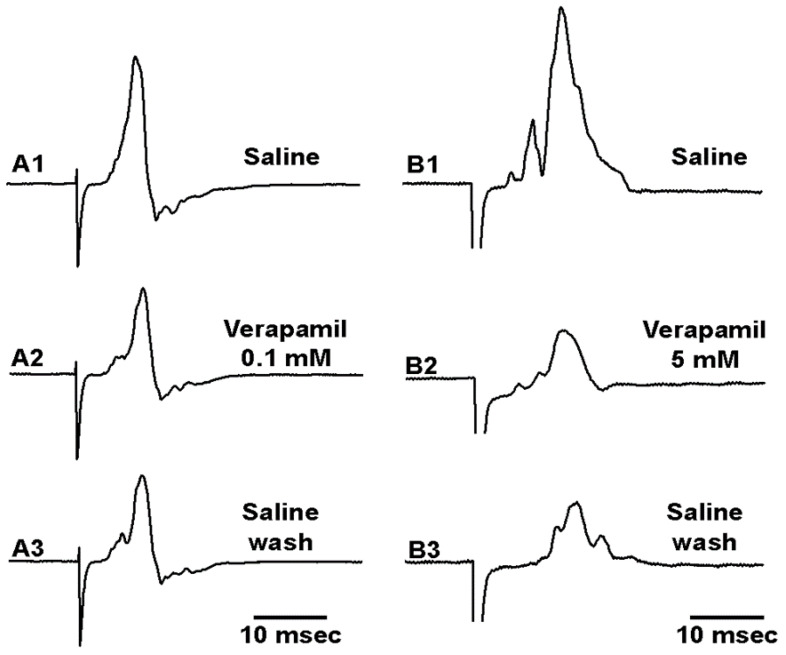
Representative effects of verapamil on the compound action potentials (CAPs) of the walking leg nerve. The CAPs in saline (**A1**,**B1**) and after 20 min of exposure to 0.1 mM (**A2**) or 5 mM (**B2**) of verapamil showed some depression for both concentrations. (**A3**,**B3**) Removal of verapamil with fresh saline did not fully recover the amplitude of the CAPs.

**Figure 11:**
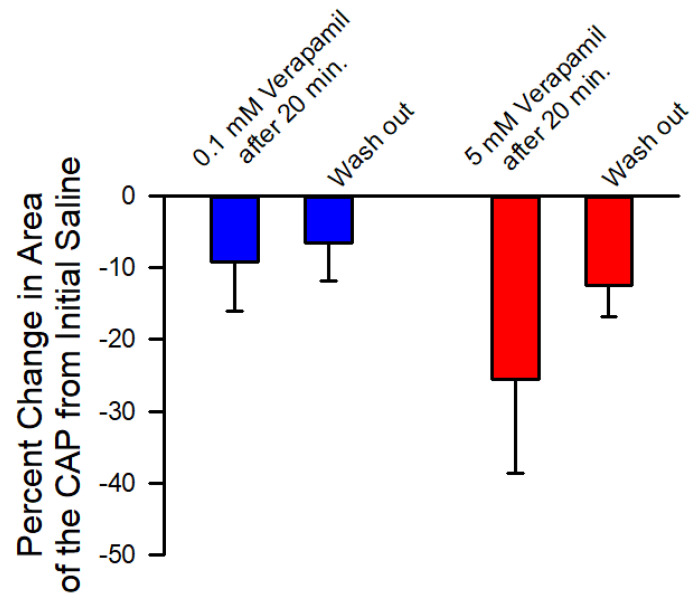
The percent change in the areas of the trace for the compound action potentials (CAPs) in saline and after 20 min of exposure to 0.1 mM or 5 mM of verapamil. The percent change in the area of the CAPs from initial saline to the wash-out is also shown. Some depression still occurred even after washout for both concentrations. (*p* < 0.05; paired T-Test; N = 6 for each concentration from initial saline to after 20 min of exposure to verapamil).

The neural activity was rapidly depressed in 5 mM verapamil and over time in 0.1 mM verapamil; thus, the effect on the axonal excitability was examined independent of the sensory endings in the PD organ. The effect on CAPs of the leg nerves were examined for both 0.1 mM and 5 mM. The amplitudes of the evoked CAPs were slightly depressed by exposure to 0.1 mM, but this was not an immediate effect. After 20 min of exposure, some preparations showed little change at 0.1 mM. Since the amplitudes of the CAPs were slightly dampened and appeared to spread over time, the area under the curve was used as an index for the effect of 0.1 mM and 5 mM. Two representative preparations are shown, one for the effect of 0.1 mM (Figure 10(A1–A3)) and one for 5 mM (Figure 10(B1–B3)). The initial amplitude in saline and after 20 min (Figure 10(A2,B2)) as well as after flushing the nerve with fresh saline (Figure 10(A3,B3)) are shown. The areas under the traces for the CAPs of the six preparations for 0.1 mM and the six for 5 mM were used to determine a percentage change from the initial saline. Exposure after 20 min of verapamil and percent change from initial saline to wash out of the verapamil was also examined and was shown to be significantly different (*p* < 0.05; paired T-Test; N = 6 for each concentration). A decrease in the area for the CAPs occurred in both 0.1 and 5 mM, but there was a greater change in area for 5 mM (Figure 11).

## 4. Discussion

**Figure 12:**
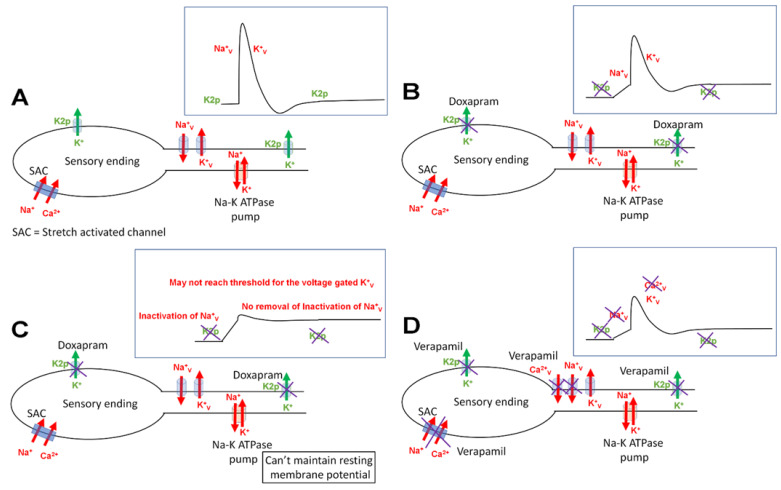
A representative model to explain the observed phenomenon with exposure to doxapram in relation to the neural activity in sensory neurons of the PD organ in a crab preparation. (**A**) A representative sensory neuron which is activated by opening stretch activated ion channels (SACs). The depolarization from activating SACs may reach the threshold to open voltage-gated Na^+^ channels (Na_v_^+^) and, subsequently, voltage-gated K^+^ channels (K_v_^+^) to allow action potentials to travel along the nerve (see inset in top right corner). The K2p channels help to maintain the resting membrane potential along with the Na^+^-K^+^ ATP dependent pump. (**B**) In the presence of doxapram, the K2p channels are blocked. (**C**) The effect of blocking the K2p channels depolarizes the neurons. A low level may bring the membrane closer to threshold to activate the Na_v_^+^ channels and produce more action potentials for the same stimulus. However, if the neuron depolarizes and cannot repolarize, the inactivation of the Na_v_^+^ channels would result in prolonged absolute refractory periods while exposed to doxapram, not allowing the neuron to be excitable. (**D**) The potential effects of verapamil are illustrated. The SACs are likely blocked rapidly upon exposure to low and high concentrations. On the axon, the voltage gated Ca^2+^ channels (Ca_v_^2+^) as well as the Na_v_^+^ may be a target given the compound action potential is slowly depressed over time, independent of the actions on the SACs in the sensory endings.

This study demonstrated that a low concentration of doxapram (0.1 mM) after 5 min of incubation resulted in hyperexcitability of the proprioceptive organ in an excised crab limb. However, a higher concentration (5 mM) after 5 min substantially decreased neural activity. The mechanism of action is likely in blocking of the K2p channels that maintain the resting membrane potential, as well as potentially the stretch activated channels in the sensory endings. It remains to be determined if K2p channels are expressed in these neurons of the crab. If these channels are present, blocking some K2p channels may depolarize the neurons to be closer to the threshold of the voltage-gated Na^+^ channels. Thus, the same sensory stimulus would more readily produce action potentials. The higher concentration of doxapram may block more K2p channels, causing depolarization without sufficient repolarization to remove the inactivation of voltage-gated Na^+^ channels. This would result in a prolonged refractory period. The proposed mechanism of action is supported by exposure to a high concentration of K^+^ which would produce a depolarized state of the neurons and would result in a prolonged state of voltage-gated Na^+^ channel inactivation until the high K^+^ is removed. Similarly, when doxapram is removed, the neural activity begins to return for the 0.1 mM exposure. This indicates the acute effects without permanent damage to the neurons. The axons were viable with 5 mM exposure to verapamil after 20 min of exposure, despite the PD organ not responding after 20 min of exposure. The stretch activated channels in the sensory endings appeared more sensitive to verapamil than channels in the axon. A working model to explain these observations is illustrated in Figure 12.

Since doxapram has an action on crab sensory neurons, this would suggest that a subtype of K2p channels similar to those described in mammals, which are doxapram-sensitive, exist in crabs as well. However, this remains to be confirmed with molecular identification, which is beyond the scope of this initial study. Doxapram is indicated to act on the TASK subtype of K2p channels [6,10]; these are acid-sensitive channels, in which low pH inhibits the function of the channel. It was demonstrated in an earlier study that low pH blocks the neural activity of the crab PD organ [26]. To date, there are no reports as to the types or number of K2p channels represented in the genomes of crustaceans. However, 15 subtypes have been identified in mammal genomes and 11 in the fruit fly *Drosophila* [32,33].It is likely that there are similar subtypes in crustaceans as in insects since they share chordotonal organs of similar anatomical structure used to monitor joint movements, as well as other physiological similarities. It would be of interest to know if multiple subtypes of K2p channels are expressed within a single cell. A high concentration of 5 mM of doxapram did not completely block the neural activity in the crab axons. This would indicate that the membrane potential did not depolarize to zero, and that other non-doxapram sensitive K2p channels are potentially helping to maintain a membrane potential to allow the neurons to remain active. Screening more organisms, from plants to animals, for types of K2p channels would pave the way for pharmacological and physiological studies to examine functional significance.

Invertebrate models can aid in screening novel pharmacological agents once research better understands the similarities and differences among organisms. This study has shown that doxapram, which is used clinically, has an action on crab neurons. It would be of interest to screen other known agonists and antagonists of K2p channels that are used in mammals on this model preparation as well as other invertebrate models such as crayfish (a freshwater crustacean) or *Drosophila* (insects). For example, verapamil (10 µM) is known to inhibit the K2p channel 18.1 [34].

In examining various pharmacological agents on the chordotonal organ, one needs to consider other channels in addition to the K2p channels. Verapamil is an L-type Ca^2+^ channel blocker [30,31]. Since the stretch-activated channels in the sensory endings of the neurons of the PD organ are blocked by Gd^3+^ [35], the channels may be stretch-activated Ca^2+^ channels which could also be blocked by verapamil. The molecular subtype of the stretch-activated channels associated with sensory neurons has yet to be identified in chordotonal organs of crustaceans [36]. The neural activity was silenced right away when bending the PD joint and exposing the preparation to verapamil (5 mM); however, when removing the sensory endings and evoking electrical activity in the nerve, the evoked CAPs took time to show depression in amplitude and upon flushing the exposed nerve the amplitude of the CAPs was able to partially return. The electrical activity was not able to regainwhen the PD organ was intact and initiating neural activity by displacement of the PD organ with joint movement. It would appear that verapamil rapidly blocked stretch-activated channels, and the compound was not able to be removed readily. In addition to verapamil potentially blocking stretch activated channels in the sensory ending, it also appeared that the voltage-gated Na^+^ channels in the axon are blocked, since the amplitude of the CAPs slowly decreases in amplitude and can return with flushing the preparation with fresh saline without verapamil. However, if the K2p channels are blocked in the axon, then the nerve may slowly be depolarized over time during the evoked responses. The voltage-gated Na^+^ channels would then be in a state of inactivation, because the membrane potential is unable to hyperpolarize enough to remove the inactivation. Flushing away the verapamil allows the membrane potential to return to a negative state to rejuvenated voltage-gated Na^+^ channels. A variable needing to be considered is that the action potential in these crab neurons may have a Ca^2+^ component, as with peripheral neurons in crayfish [37,38]. The action potential becomes narrower when the influx of Ca^2+^ is reduced. In central neurons within crayfish, verapamil can alter the shape of the action potential [39]. If verapamil blocks the voltage-gated channels in axons, then the calcium-activated potassium conductance would also be reduced. Calcium-activated potassium channels are known to be present in crayfish neurons [38], If this occurs, the frequency of activity could decrease as the neuron would not reset to baseline as quickly and remove inactivation of the sodium channels. Thus, single channel recordings of the SACs as well as the subtype of ion channels in the axon are needed to better understand the mechanism of action for verapamil on this preparation.

In order to examine reproducibility in the observations and avoid potential bias by a given investigator, five groups of the total 17 students investigated the same procedures as three displacements in saline and 3 more after initial exposure to doxapram (5 mM) and after 5 min of incubation in doxapram followed by two saline flushes of the preparation. As shown in the Appendix A, the overall trends proved consistent. The experiments performed with the course participants may not have been as consistent in the rate of movements as using one individual for all the data sets presented in the Results. University policies indicated that the experiments performed in the classroom had to be conducted within a fume hood due to low airflow in the teaching laboratory.

Since it is now common practice to provide raw data sets with publications, it is important to understand how the data were analyzed, as differences in interpretation could occur. This is particularly relevant when using automated analysis procedures provided by commercial software. It is suggested to use an approach of measuring the spikes which occur outside two times the level of the background noise in the types of recordings performed herein. Issues faced with data analysis are described in the Appendix A. A detailed explanation in analysis with the software used in this study is provided in an earlier study [29]. When following the details provided in Tanner et al., [29] the errors shown can be avoided.

## Figures and Tables

**Figure A1:**
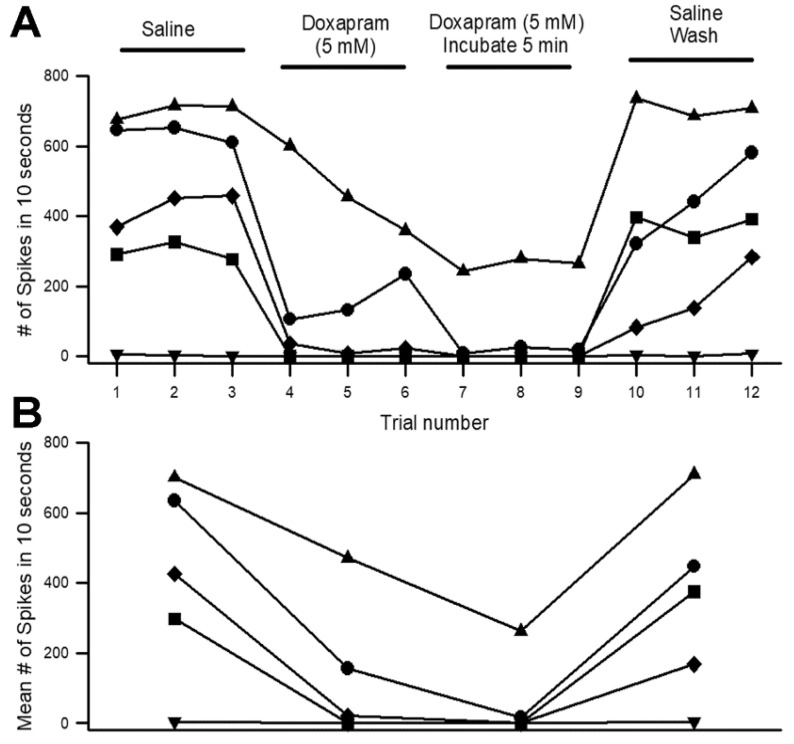
The activity obtained from the PD nerve for five different groups within a university course for the effect of doxapram (5 mM). (**A**) The number of spikes were measured in the same way and by the same person who measured all the traces independently of the student participants for consistency in analysis. (**B**) The number of spikes in each of the three trials was averaged and graphed in the same manner as in (**A**), which allows an easier view of the overall effects.

**Figure A2:**
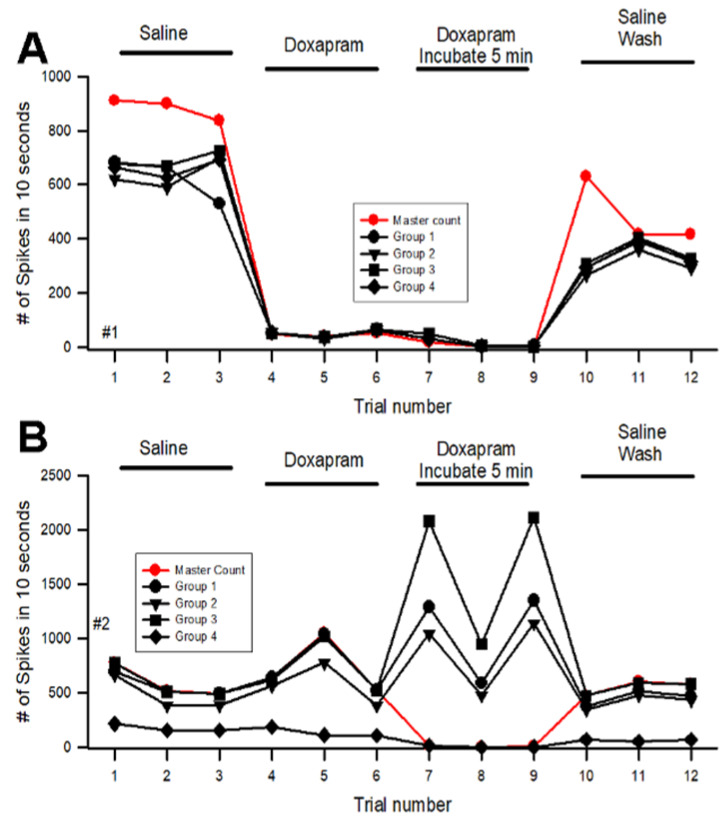
Four groups of different participants, with two people per group, analyzed the same data sets (A #1 and B #2). (**A**) The four groups who analyzed the #1 data set fits well with the analysis from a master in analysis, whose counts are indicated by the red line. The master analyzed all data sets in the study. (**B**) The participants deviated from the master in analysis for three of the four groups in the condition where the doxapram had been incubated for 5 min. The other experimental conditions followed a similar trend as that of the master.

**Figure A3:**
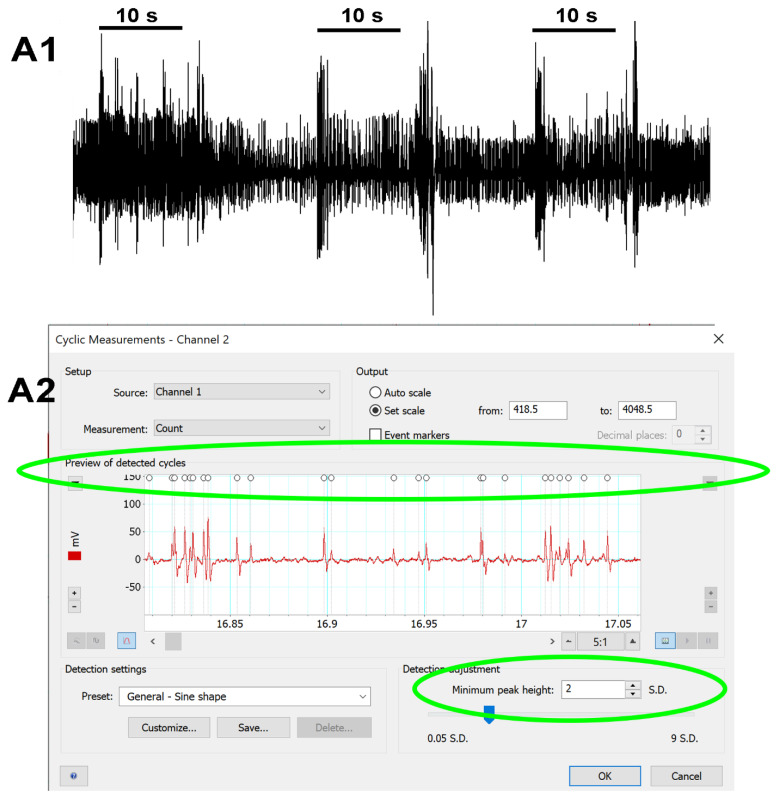

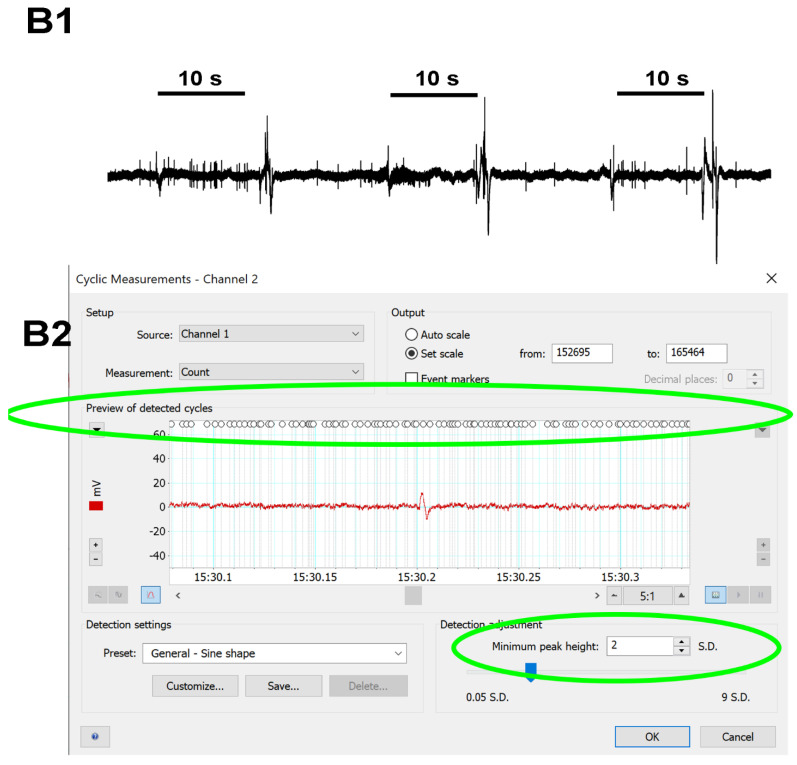
Analysis of data with the same automated measure. Two traces are provided: three trials in saline (**A1**) and the three trials in doxapram after 5 min of incubation (**B1**). The automated analysis is set to 2 SD (standard deviations of the mean in the traces; see small green ellipse in (**A2**,**B2**)) for both (**A1**,**B1)**, but it produces differences in what is detected, as indicated in the large green ellipse in (**A2**,**B2**). The open circles inside the ellipse indicate what deflections in the trace are counted as spikes. Since there is little neural activity in (**B1**), the noise of the trace is being detected as spikes, producing an artificially high number of spikes.

## Data Availability

All data are available in the manuscript and are available upon request.

## Appendix A

### Appendix A.1. Reproducibility in the Effects of Doxapram with a University Course

Five groups with a total of 17 students investigated the same general procedures as reported in the main study. Each group displaced their crab leg three times in saline, then three times after initial exposure to doxapram (5 mM), then three times after five minutes of incubation in doxapram, and finally three times after two saline washes. The activity profiles for the five preparations performed with the course are shown in Figure A1.

Conditions varied depending on how the joint was moved and the extent of flushing solutions around the dish in which the preparation was placed. Preparations were dissected prior to the course starting. Some were dissected 3 to 4 h before being examined; however, the saline bath was exchanged with fresh saline about every hour prior to experimentation. In addition, due to requirements of the university chemical safety committee, the experiments needed to be performed within a fume hood, which produced some vibration and thus activity of the sensory neurons.

### Appendix A.2. Reproducibility in Analysis of Given Data Sets

Two data sets were given to four different groups of participants without knowing the compound provided or its concentration. Thus, this was a blind study in the analysis. Each group consisted of two participants working together. An explanation of how to count the number of spikes within 10 s of the stimulus was provided. The results were compared to a person deemed as a master in analysis since this person analyzed all the data sets for the whole study. The two given data sets are shown as A (#1) and B (#2) within Figure A2. Spike counts in the A data set were relatively consistent for the four different groups of participants, and the overall trends were similar. The data set shown in Figure A2B proved to be challenging to the course participants using the automated software for analysis. One of the four groups had a similar trend as the master.

Further investigation and discussion with the class revealed the reasoning for the discrepancy. Upon analysis of the data in the initial three saline trials, the standard deviation of the mean value of the trace was set to a value of 2 (Figure A3(A1)). This setting was ideal to detect the spikes above the background noise. However, since the number of spikes decreased in the set of three trials after the 5-min incubation in doxapram (5 mM) (Figure A3(B1)), the automated software picked up noise, as it was still determining it to be above the mean by two times the standard deviation of the trace. In this case, there were no large spikes, so small deflections in the noise were counted in the automated detection process (Figure A3(A2,B2)). A detailed explanation of these issues in analysis and how to correct for the errors with the software is provided in an earlier study [29]. Since the baseline moves in some cases while moving the joint and the spikes along with the baseline are easily seen visually, but if a set threshold is used to detect spikes, errors will arise as the baseline moves and crosses a threshold.
